# Too Much of a Good Thing: How Ectopic DNA Replication Affects Bacterial Replication Dynamics

**DOI:** 10.3389/fmicb.2020.00534

**Published:** 2020-04-15

**Authors:** Aisha H. Syeda, Juachi U. Dimude, Ole Skovgaard, Christian J. Rudolph

**Affiliations:** ^1^Department of Biology, University of York, York, United Kingdom; ^2^Division of Biosciences, College of Health and Life Sciences, Brunel University London, Uxbridge, United Kingdom; ^3^Department of Science and Environment, Roskilde University, Roskilde, Denmark

**Keywords:** replication, transcription, recG gene, termination of DNA replication, ectopic replication origins, bacterial replication dynamics, 3′ exonuclease

## Abstract

Each cell division requires the complete and accurate duplication of the entire genome. In bacteria, the duplication process of the often-circular chromosomes is initiated at a single origin per chromosome, resulting in two replication forks that traverse the chromosome in opposite directions. DNA synthesis is completed once the two forks fuse in a region diametrically opposite the origin. In some bacteria, such as *Escherichia coli*, the region where forks fuse forms a specialized termination area. Polar replication fork pause sites flanking this area can pause the progression of replication forks, thereby allowing forks to enter but not to leave. Transcription of all required genes has to take place simultaneously with genome duplication. As both of these genome trafficking processes share the same template, conflicts are unavoidable. In this review, we focus on recent attempts to add additional origins into various ectopic chromosomal locations of the *E. coli* chromosome. As ectopic origins disturb the native replichore arrangements, the problems resulting from such perturbations can give important insights into how genome trafficking processes are coordinated and the problems that arise if this coordination is disturbed. The data from these studies highlight that head-on replication–transcription conflicts are indeed highly problematic and multiple repair pathways are required to restart replication forks arrested at obstacles. In addition, the existing data also demonstrate that the replication fork trap in *E. coli* imposes significant constraints to genome duplication if ectopic origins are active. We describe the current models of how replication fork fusion events can cause serious problems for genome duplication, as well as models of how such problems might be alleviated both by a number of repair pathways as well as the replication fork trap system. Considering the problems associated both with head-on replication-transcription conflicts as well as head-on replication fork fusion events might provide clues of how these genome trafficking issues have contributed to shape the distinct architecture of bacterial chromosomes.

## Introduction

While eukaryotic cells typically contain multiple linear chromosomes, the bacterial models studied in most detail early on, such as *Escherichia coli* and *Bacillus subtilis*, have a single chromosome with a size of roughly 5 Mbp that forms a covalently closed circle. The improved ability to sequence whole genomes has revealed considerable variations. For example, *Mycoplasma genitalium*, a sexually transmitted pathogen that can cause non-gonococcal urethritis, is one of the smallest prokaryotes capable of independent replication with a genome size of 0.58 Mbp and less than 500 genes (Taylor-Robinson and Jensen, 2011; Gnanadurai and Fifer, 2020). In strictly opportunistic or symbiotic bacteria, genomes can be even smaller: the symbiotic bacterium *Carsonella ruddii* carries a single circular chromosome containing 0.159 Mbp and is predicted to encode 182 genes (Nakabachi et al., 2006). The genome of the myxobacterium *Sorangium cellulosum*, on the other hand, contains just over 13 Mbp and is predicted to encode 9,367 coding sequences (Schneiker et al., 2007). Overall, protein-coding density of bacterial genomes is with 85–90% high (McCutcheon and Moran, 2011) and the correlation between genome size and the number of genes is surprisingly constant (Touchon and Rocha, 2016).

Many of the extensively studied bacterial models are haploid. In *E. coli*, overlapping cell cycles in fast growing cells allow an increase in genome equivalents and stationary cells contain only a single copy of the chromosome. In contrast, many other bacterial species carry multiply copies of the chromosome. *Deinococcus radiodurans* carries between four and 10 genome equivalents (Hansen, 1978), and the presence of multiple copies is thought to be one contributor to its extreme radiation resistance (Minton and Daly, 1995; Timmins and Moe, 2016). Bacteria such as *Azotobacter vinelandii* can carry up to 80 chromosome copies per cell under fast growth conditions (Nagpal et al., 1989), and tens of thousands of copies were reported for the large bacterium *Epulopiscium* (Mendell et al., 2008).

While the presence of multiple chromosome equivalents is relatively common, the presence of more than one type of chromosome is less frequent, found in about 5% of bacterial species investigated so far (Touchon and Rocha, 2016). Examples are *Vibrio cholerae* and close relatives of *Vibrio*, which usually carry two circular chromosomes (Touchon and Rocha, 2016), while *Paracoccus denitrificans*, a gram-negative soil bacterium, carries three different circular chromosomes (Winterstein and Ludwig, 1998).

While the majority of bacterial chromosomes form covalently closed circles, some bacterial species carry linear chromosomes or even a mix of circular and linear chromosomes. For example, *Agrobacterium tumefaciens* carries one circular and one linear chromosome, as well as two very large plasmids (Nester, 2015). Linear chromosomes are frequently found within the *Actinomycetales*, which includes the genus *Streptomyces* (Kirby, 2011). The normally circular *E. coli* chromosome can also be artificially linearized using the telomere system of bacteriophage N15, and the resulting cells grow stably without any observed ill effect (Cui et al., 2007; Rudolph et al., 2013).

## The Bacterial Replichore Arrangement

**FIGURE 1:**
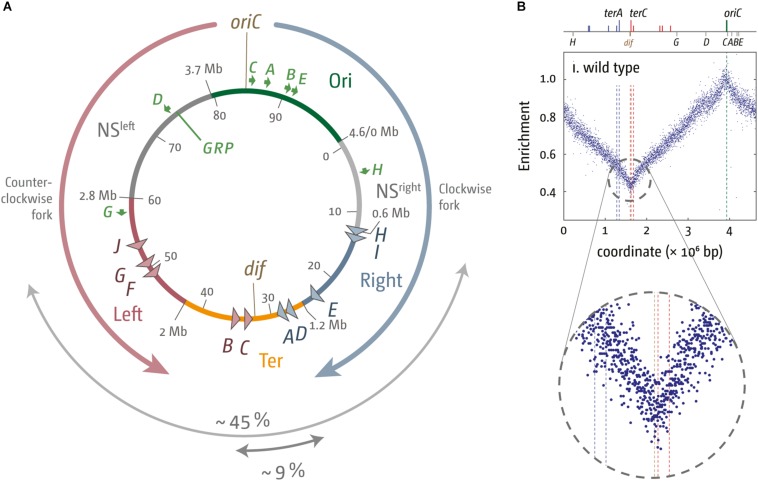
Chromosome structure and replication dynamics in *Escherichia coli*. **(A)** Schematic representation of the *E. coli* chromosome. Two replication forks are initiated at the origin (*oriC*) move in opposite directions along the DNA and eventually approach one other and fuse within the terminus region diametrically opposed to *oriC*. A replication fork trap is formed in the terminus region via terminator sequences (*terA–J*) which are arranged as two opposed groups, with the red terminators oriented to block movement of the clockwise replication fork and the blue terminators oriented to block the anticlockwise fork. The large gray arrow highlights the total spanned area covered by ter sites, while the core termination area, defined by the four innermost ter sites, is marked by a small gray arrow. The chromosomal locations for *oriC* and the dif chromosome dimer resolution site are marked. The location of rrn operons, which are highly transcribed particularly under fast growth conditions, are shown by green arrows, with the arrow pointing in the direction in which transcribing RNA polymerase molecules travel. “GRP” indicates the location of a cluster of genes encoding ribosomal proteins, almost all of which are transcribed co-directionally with replication. Chromosomal macrodomains Ori, NS^right^, NS^left^, Right, Left, and Ter are shown as described in Duigou and Boccard (2017) and domain boundaries given in Mbp. Numbers on the inside are the minutes of the standard genetic map (0–100 min). **(B)** Marker frequency analysis of wild type *E. coli* cells. The number of reads (normalized against reads for a stationary phase wild type control) is plotted against the chromosomal location. A schematic representation of the *E. coli* chromosome showing positions of *oriC* (green line) and ter sites (above) as well as dif and rrn operons A–E, G, and H (below) is shown above the plotted data. The MFA raw data were taken from Rudolph et al. (2013) and re-plotted to allow changes the scale of the plots, if necessary, and to highlight specific schematic features of the *E. coli* chromosome. A magnified view of the replication profile in the termination area is shown in the enlarged circle.

Despite these considerable variations, the replichore arrangements of most bacterial genomes are straightforward. While replication of the multiple linear chromosomes in eukaryotic cells is initiated at hundreds or even thousands of origins (Leonard and Méchali, 2013), initiation sites in bacteria are restricted to a single origin per chromosome (*oriC*) (Gao and Zhang, 2008; Gao, 2015). For a bacterium such as *E. coli*, this means that the number of replisomes is restricted to two, which are recruited at the origin and proceed in opposite directions until they eventually fuse opposite the *oriC* (Masters and Broda, 1971; Prescott and Kuempel, 1972; Dimude et al., 2016). Thus, each chromosomal half or replichore (Blattner et al., 1997) is replicated by one fork in a defined directionality (Figure 1A). DNA replication is successfully completed once every single base pair of the chromosome is duplicated with high accuracy. However, daughter chromosomes will remain interlinked until they are resolved through post-replicative processing (Lesterlin et al., 2004; Reyes-Lamothe et al., 2012), a process that is coordinated both temporally and spatially with septum formation at mid-cell (Reyes-Lamothe et al., 2012; Zaritsky and Woldringh, 2015).

In *E. coli*, the replichore arrangement results in certain asymmetric features of the chromosomal halves. For example, the leading and lagging strands show a nucleotide composition bias, with G being overrepresented in the leading strand (Wu and Maeda, 1987; Lobry, 1996; Blattner et al., 1997). The contributions from transcription and replication toward this bias is still under debate (Francino et al., 1996; Rocha et al., 2006; Chen et al., 2016), but replication and replication-linked processes, such as cytosine deaminations, which were shown to occur preferentially in the lagging strand (Bhagwat et al., 2016), clearly contribute. The compositional bias results in a sharp transition both at the origin and the terminus near the dif dimer resolution site (Wu and Maeda, 1987; Lobry, 1996; Blattner et al., 1997; Lobry and Louarn, 2003). In addition, the KOPS 8-mer (FtsK Orienting Polar Sequences) is asymmetric, with a preference of pointing toward the dif chromosome dimer resolution site. This allows not only binding, but also the directional movement of Ftsk, which is essential for the unlinking of chromosome dimers that can arise as a result of an odd number or recombination events (Bigot, 2005; Levy et al., 2005; Barre, 2007; Sherratt et al., 2010).

Higher order genome organization appears to correlate to some extent with the replichore arrangement. In initial experiments it was observed that relatively large regions of the chromosome colocalize *in vivo*, leading to the suggestion of the existence of one macro domain that contains the origin area and a second macrodomain that contains the terminus area of the chromosome (Valens et al., 2004; Verma et al., 2019). The macrodomain structure of the chromosome was further investigated with fluorescence-microscopy and recombination-based approaches as well as, most recently, with chromosome conformation capture methods (3C), leading to the idea that the *E. coli* chromosome is divided into four macrodomains (Ori, Ter, Left, and Right) as well as two more flexible and non-structured regions, NS-L and NS-R, that flank the Ori macrodomain (Liu et al., 2010; Duigou and Boccard, 2017; Verma et al., 2019; Figure 1A).

## The Termination Area in *Escherichia Coli*

One peculiarity of the termination area both in *E. coli* and *B. subtilis* is the ability to restrict fork movement via a “replication fork trap,” a series of protein-DNA complexes that are asymmetric. An approaching fork coming from one direction can displace the bound protein and continue to traverse the chromosome, while a fork coming from the other direction will be paused and unable to proceed past the block for some time. The short DNA sequences involved are called terminator, or ter, sequences. In *E. coli* each ter sequence can be bound by a single Tus protein (terminus utilization substance), while in *B. subtilis* ter sequences are bound by an Rtp (replication termination protein) dimer. Both *E. coli* and *B. subtilis* are similar in that the ter sequences are positioned to form two opposed groups that allow replication fork complexes to enter but not exit the termination region. However, the overall size of the termination area differs significantly: while in *E. coli* ter sequences are distributed over >40% of the chromosome (Figure 1A), the spread is much narrower in *B. subtilis* (<10%). However, in normally growing *E. coli* cells, only the four inner-most ter sites, *terC* and *terB* on one side and *terA* and *terD* on the other, are substantially involved in the arrest of DNA replication (de Massy et al., 1987; Hill et al., 1987; Duggin and Bell, 2009). Thus, these four sites are considered to be the primary fork trap, and with about 9% their spread is similar to the spread of ter sites in the *B. subtilis* chromosome (Figure 1A).

In *E. coli* MG1655, *terC* is generally the first ter/Tus complex to be encountered by a replisome (Duggin and Bell, 2009). *terC* is located almost directly opposite the origin and will arrest the replisome traversing the chromosome in clockwise orientation (Figure 1A). The second innermost ter site is *terA*, which is located in a slightly more asymmetric position (Figure 1A). The outer terminators are probably used only rarely (Griffiths and Wake, 2000; Duggin and Bell, 2009). However, it is important to note that ter/Tus complexes are not systematically involved in replication termination. This was already shown by early labeling experiments (Bouché et al., 1982) and supported more recently by high-resolution replication profiles established via deep sequencing.

High-resolution replication profiles can be generated from marker frequency analyses (MFA) by deep sequencing (Skovgaard et al., 2011; Rudolph et al., 2013; Müller et al., 2014). MFA is generated by plotting the ratio of uniquely mapped sequence reads per 1 kb window in a replicating sample relative to a non-replicating control (stationary phase wild type cells). The replication profile for rapid growing wild type cells shows the location of *oriC* as a clear maximum, while a minimum in the termination area shows the most common fork fusion point (Skovgaard et al., 2011; Rudolph et al., 2013; Ivanova et al., 2015).

The fact that replication profiles show a distinct V-shaped low point (Figure 1B) suggests that the majority of fork fusions in *E. coli* take place near the arithmetic mid-point. Indeed, we observed that the low point of the replication profiles in the presence and absence of a functional fork trap was in the same location (Rudolph et al., 2013; Ivanova et al., 2015; Dimude et al., 2016), suggesting that both replisomes traverse their replichores with similar speeds and fuse freely within the innermost ter sites. It appears that the fork trap is only involved in termination if one replisome is delayed at an obstacle on its way through the replichore (Duggin et al., 2008; Duggin and Bell, 2009).

A recent analysis from Galli et al. (2019) has shown that Tus-related sequences are found in most *Enterobacteriales*, in the *Pseudoalteromonas*, and in most *Aeromonadales*. In contrast, RTP-related sequences are restricted to a subgroup of the *Bacillales* (Galli et al., 2019). Indeed, sequence analysis suggests that a replication fork trap is absent in many bacterial species. This was experimentally demonstrated for the two circular *Vibrio cholerae* chromosomes (Galli et al., 2019). Similarly, no specific termination-related pause sites have been identified in eukaryotes and archaea, even though multiple replication origins per chromosome result in a much higher number of fork fusions. It appears that replication effectively terminates at random locations between origins (Duggin et al., 2011; Hawkins et al., 2013; Samson et al., 2013; Gambus, 2017).

The absence of any significant sequence or structural similarity of the components of the fork trap in *E. coli* and *B. subtilis* indicates that fork trap systems have evolved via convergent evolution (Neylon et al., 2005). If this is the case, then the system would be expected to have an important physiological function. However, early studies suggested that the inactivation of the fork trap both in *B. subtilis* and *E. coli* has very little effect on growth rate and cell morphology (Iismaa and Wake, 1987; Roecklein et al., 1991), suggesting that our understanding of the physiological role of the termination area is incomplete. We will explore possible roles of the replication fork trap later in this review.

## Coordinating Replication and Transcription

The combination of a single point of replication initiation with a fork trap mechanism enforces a strong directionality of replication in wild type cells, as each replichore is replicated in a defined orientation under normal conditions. It was suggested that this directionality might be advantageous (Brewer, 1988; French, 1992; Dimude et al., 2016). Replication and transcription move with very different speeds, as transcription is significantly slower than DNA replication (Vogel and Jensen, 1994; Dennis et al., 2009), and, given that both processes utilize the same template, conflicts are unavoidable. Indeed, highly transcribed genes were found to be preferentially located on the template for the leading strand in a number of bacterial species, resulting in the co-directional movement of replisomes and transcribing RNA polymerase complexes (Brewer, 1988; McLean et al., 1998; Rocha and Danchin, 2003; Evertts and Coller, 2012). In *E. coli*, global co-orientation is only just under 55%, but over 90% of genes encoding ribosomal proteins, which are particularly highly transcribed, show co-directionality of replication and transcription (Brewer, 1988; McLean et al., 1998; Figure 1A). A higher general co-orientation was observed in other bacteria, with more than 70% of genes being transcribed co-directionally with replication in *B. subtilis* and *Mycoplasma pneumonia*, with virtually all genes that code for ribosomal proteins being transcribed co-directionally with replication (McLean et al., 1998).

The co-directionality of highly transcribed genes and DNA replication indicates head-on encounters of replisomes with transcribing RNA polymerase complexes are particularly problematic (French, 1992; Rudolph et al., 2007a; Kim and Jinks-Robertson, 2012; McGlynn et al., 2012; Merrikh et al., 2012), even though any encounter can interfere with ongoing DNA replication (Merrikh et al., 2011; Lang and Merrikh, 2018). Indeed, it was shown in both *E. coli* and *B. subtilis* cells that replication of a highly transcribed rrn operon in an orientation opposite to normal caused significant problems (Wang et al., 2007; Boubakri et al., 2010; Srivatsan et al., 2010; De Septenville et al., 2012; Million-Weaver et al., 2015).

In eukaryotic cells, replication–transcription encounters are expected to cause similar problems. However, initially the analysis of replication and transcription directionality in human cells has revealed little overall bias, suggesting that the orientation of open reading frames might be effectively random (Necsulea et al., 2009; Hyrien, 2015), perhaps with the exception of yeast, in which a replication barrier prevents forks from entering highly transcribed ribosomal DNA repeats in a head-on orientation (Hyrien, 2000; Mirkin and Mirkin, 2007; Evertts and Coller, 2012). This view has recently changed. A recent study showed a preference for replication initiation sites in human cells to occur in the immediate vicinity of transcription start sites, while termination of synthesis occurs at the 3′ end of genes, highlighting that the same fundamental principle of co-directionality applies in human cells (Chen et al., 2019).

## Constraints of the Bacterial Replichore Arrangement

While there is a certain esthetic beauty to the straightforward bacterial replichore arrangement, this system also imposes significant constraints. If replication is initiated exclusively at a single origin, then the ability of fast growth is directly linked to the speed of chromosome duplication. Indeed, the speed of replication in *E. coli* is 650–1000 nt × s^–1^ (Pham et al., 2013), which is about 20 × faster than DNA replication in human cells (Méchali, 2010). The use of 30,000–50,000 origins in human cells can compensate for slow speed and the longer duplication time of the larger genome, and indeed, in *Xenopus laevis* and *Drosophila melanogaster*, origins are activated at very short intervals during early embryonic development (Méchali, 2010). Bacteria such as *E. coli* have to utilize overlapping rounds of DNA synthesis in order to achieve a cell duplication period that is shorter than the time required to duplicate the entire chromosome (Dewachter et al., 2018). Chromosome duplication is completed in approximately 40 min, but cells can divide every 20 min in rich medium that allows overlapping rounds of DNA replication. Indeed, under conditions where progression of ongoing DNA synthesis is blocked by DNA lesions while initiation at *oriC* can still take place, a temporary cell division period of <15 min is observed once the lesions have been eliminated, allowing all initiated forks rapidly to generate complete chromosomal copies (Rudolph et al., 2007b, 2010b).

The presence of a replication fork trap as part of the chromosome architecture in bacteria would appear to be particularly problematic in the face of obstacles to DNA replication. While replication in *E. coli* is very fast and accurate, progression of synthesis will always encounter obstacles, including stable protein-DNA complexes, secondary structures, a variety of DNA lesions and other problems (Cox, 2001; McGlynn et al., 2012; Merrikh et al., 2012). If duplication of a chromosome is restricted to two replication forks and a replication fork trap is present, such obstacles can have potentially disastrous consequences. If one fork is permanently blocked, a replication fork trap will prevent it being rescued by the second fork, as this fork will also be blocked (Dimude et al., 2016). We believe that this particular problem explains in part why replication restart proteins such as PriA are so prominent in bacteria (Dimude et al., 2016), as these proteins are essential for the re-recruitment of functional replisomes following the removal of obstacles of damage (Gabbai and Marians, 2010; Windgassen et al., 2018).

Why are bacterial chromosomes exclusively replicated using a single origin if this scenario can be problematic? In eukaryotic cells, under-replicated stretches of DNA can trigger activation of “dormant” origins that aid the completion of DNA synthesis if progression of early forks is delayed by obstacles or damage (Blow et al., 2011; Courtot et al., 2018). Whilst archaea predominantly carry circular chromosomes, at least some species utilize multiple origins to replicate their genomes (Lundgren et al., 2004; Wu et al., 2014). Thus, the consistent use of a single origin in bacteria may seem surprising, especially as gross chromosomal rearrangements can occur relatively frequently (Umenhoffer et al., 2017).

## Introducing a Second Origin Into the *E. Coli* Chromosome

**FIGURE 2:**
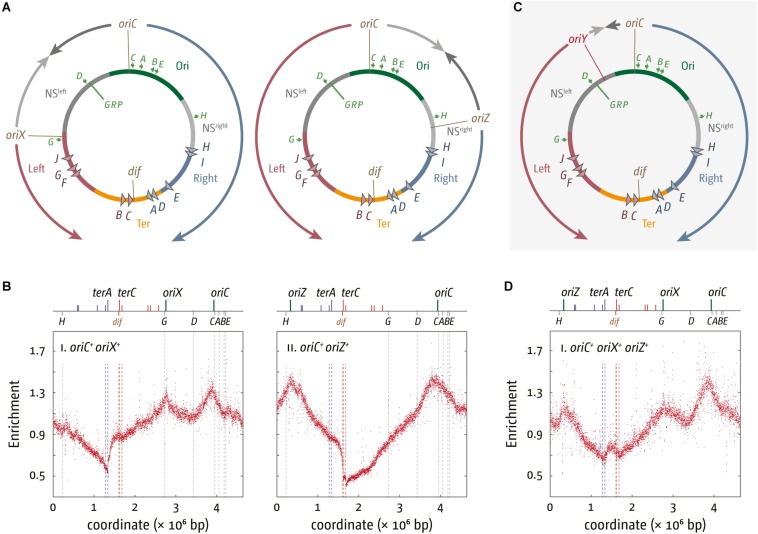
Chromosome structure and replication dynamics in *E. coli* cells with additional ectopic replication origins. **(A)** Integration sites of 5 kb *oriC* fragments into pheA upstream of the rrnG operon, termed oriX, and near the lacZYA operon, termed oriZ (Wang et al., 2011; Ivanova et al., 2015; Dimude et al., 2018b). All genetic and structural elements shown are as described in Figure 1. **(B)** Marker frequency analysis of *E. coli oriC*^+^ oriX^+^ and *oriC*^+^ oriZ^+^ cells. The number of reads (normalized against reads for a stationary phase wild type control) is plotted against the chromosomal location. A schematic representation of the *E. coli* chromosome showing positions of *oriC*, oriX and oriZ (green lines) and ter sites (above) as well as dif and rrn operons A–E, G, and H (below) is shown above the plotted data. The MFA raw data were taken from Dimude et al. (2018b) and re-plotted to allow changes the scale of the plots, if necessary, and to highlight specific schematic features of the *E. coli* chromosome. **(C)** Integration site of a 5 kb *oriC* fragment, termed oriY, into malT, upstream of the rrnD operon. See text for details. **(D)** Marker frequency analysis in *E. coli oriC*^+^ oriX^+^ oriZ^+^ cells. The number of reads (normalized against reads for a stationary phase wild type control) is plotted against the chromosomal location. A schematic representation of the *E. coli* chromosome showing positions of *oriC*, oriX, and oriZ (green lines) and ter sites (all above) as well as dif and rrn operons A–E, G, and H (all below) is shown above the plotted data. The MFA raw data were taken from Dimude et al. (2018b) and re-plotted to allow changes the scale of the plots, if necessary, and to highlight specific schematic features of the *E. coli* chromosome.

Given the multiple origins per chromosome in archaea and eukaryotes, researchers have asked whether multiple origins can be utilized in bacterial chromosomes. The Sherratt lab was able to integrate a 5 kb *oriC* fragment near the lac operon into the *E. coli* chromosome, roughly in the middle of the right-hand replichore (Wang et al., 2011). To distinguish this origin from the native *oriC*, this origin was termed oriZ even though the sequence is identical to *oriC* (Figure 2A). Cells carrying both *oriC* and oriZ, which we will refer to as *oriC^+^* oriZ*^+^* cells, were reported to have doubling times similar to wild-type cells, and fluorescence microscopy confirmed that both origins are active and fire simultaneously (Wang et al., 2011).

In line with the fluorescence microscopy data (Wang et al., 2011) the replication profile of *oriC^+^* oriZ*^+^* cells shows a second maximum at the location of oriZ and an additional and ectopic local minimum between *oriC* and oriZ (Figure 2B), indicative of a second fork fusion point (Rudolph et al., 2013; Ivanova et al., 2015). However, the primary minimum of the replication profile shows a distinct step in between *terA* and *terB/C* in *oriC*^+^ oriZ^+^ cells, rather than a V-shape. As oriZ is roughly in the middle of the right-hand replichore, forks initiated at oriZ and traversing toward the termination area only have to duplicate 1/4 of the chromosome before they reach the fork trap area, while the fork initiated at *oriC* and proceeding counterclockwise has to replicate the entire replichore. Thus, within a randomly growing population there will be significantly more cells in which forks coming from oriZ will get trapped at *terC* and subsequent ter sites until the second fork reaches this area, resulting in the defined “step” between *terA* and *terC* (Figure 2B).

In the initial analysis, the doubling time of *oriC*^+^ oriZ^+^ and wild type cells was found to be similar (Wang et al., 2011). However, when we measured the doubling times for MG1655 and *oriC*^+^ oriZ^+^ constructs in direct comparison, we found that *oriC*^+^ oriZ^+^ cells grew slightly slower in two independent studies (∼21 min in comparison to ∼20 min in wild type cells) (Ivanova et al., 2015; Dimude et al., 2018b).

We also integrated the same 5 kb *oriC* fragment roughly into the middle of the left-hand replichore, which resulted in the generation of *oriC*^+^ oriX^+^ cells (Figure 2A; Dimude et al., 2018b). The replication profile of these cells proved very similar to the profile observed in *oriC*^+^ oriZ^+^ cells (Figure 2B). MFA analysis confirmed that oriX was active and suggests that both *oriC* and oriX fire simultaneously in the majority of cells, a result confirmed via fluorescence microscopy (Dimude et al., 2018b). Replication profiles of *oriC*^+^ oriX^+^ cells showed the same general features as the profiles from *oriC*^+^ oriZ^+^ cells, including a step in the termination area which is located at *terA* (Dimude et al., 2018b).

As already observed for *oriC*^+^ oriZ^+^ cells, we again found the doubling times for *oriC*^+^ oriX^+^ constructs to be slightly longer (∼22 min vs. ∼19.5 min for wild type cells) (Dimude et al., 2018b), providing additional confirmation that the introduction of an additional ectopic origin interferes with genome duplication and/or segregation.

While the integration of a second ectopic origin proved relatively unproblematic in both replichores aside from the mild growth defect, other attempts were less successful. The integration of a plasmid-derived origin that could be induced with IPTG at a location ∼450 kb away from *oriC* was successful, but if this origin was active, it repressed activity of *oriC* (Kouzminova and Kuzminov, 2008). In another study, integration of a shorter *oriC* fragment in two chromosomal locations, one roughly equivalent to the oriZ position while the second was closer to the termination area (1.6 Mbp), did not result in any detectable initiation at the ectopic origins (Milbredt et al., 2016). The authors suggested that origin activity might be influenced by the presence of flanking genes (Milbredt et al., 2016), which would explain why the longer 5 kb *oriC* region stretch developed in the Sherratt lab (Wang et al., 2011) proved active. However, our attempts to integrate the same 5 kb *oriC* fragment into the *malT* gene at 76.5 min, approximately 1/4 into the left-hand replichore, to generate *oriC*^+^ oriY^+^ cells (Figure 2C), proved unsuccessful. We had little difficulty getting chromosomal integrations displaying the correct antibiotic resistance. However, the oriY was not active and PCR analysis of two independent oriY constructs showed that the *oriC* core elements were either truncated or completely absent (Dimude et al., 2018b). The difference of the truncations observed suggests that they are spontaneous mutations, arising perhaps because of a toxicity caused by an active origin being integrated in this precise location. Given that the integration of the antibiotic resistance marker occurred without any problem, it appears that the integration of an ectopic sequence in this location is unproblematic.

## Introducing Three Origins Into the *E. Coli* Chromosome

We went on to generate an *oriC*^+^ oriX^+^ oriZ^+^ strain with three origins, which proved unproblematic. However, the replication profile of this construct revealed a surprising detail: the peak heights of ectopic origins oriZ and oriX were reduced in comparison to the peak height of the native *oriC* (Figure 2D; Dimude et al., 2018b). This indicates that both ectopic origins, oriX and oriZ, are used less frequently than the native *oriC*, a result that contrasts with both double-origin constructs where the peak heights of the native *oriC* and the ectopic origin were very similar (Dimude et al., 2018b). Replication profiles are population-based, and for this reason allow little insight into origin usage in single cells. To directly visualize active replisomes in oriX^+^ *oriC*^+^ oriZ^+^ cells we used YPet-DnaN, a fluorescently tagged version of the β sliding clamp. Previously we observed that the signal in double-origin cells produced defined foci, as described before (Wang et al., 2011). In contrast, foci in triple-origin cells were much less defined. The analysis of foci, which are not only in close proximity but, in addition, not particularly well defined, proved rather difficult. However, we observed some cells with three separate foci, indicating that all three origins are active in these cells. However, the replication profiles clearly show a reduced activity of the ectopic origins in comparison to *oriC*, as the peak height of both ectopic origins is lower than the peak height of *oriC*. The difference in peak heights suggests that in some cells only two origins are active, but as the *oriC* peak is the highest it indicates that in these cells one of the two active origins is always the native *oriC*, whereas the ectopic origin is either oriX or oriZ.

We also observed that cultures of triple-origin cells showed an increase of cells with no foci. This could be due to a frequent failure of ongoing replication. Alternatively, it could highlight a failure to initiate replication. For example, a threshold concentration of the DnaA initiator protein is required for successful initiation (Boye et al., 2000). An increase in the number of origins will lead to an increase in the number of DnaA binding sites, which will cause a drop in the concentration of free DnaA. In a fraction of cells this drop might result in none of the origins being activated, as observed. No such effect was observed in any of the double-origin constructs (Wang et al., 2011; Ivanova et al., 2015; Dimude et al., 2018b), indicating that levels of free DnaA must be high enough to allow simultaneous initiation if two origins in the vast majority of cells. Thus, we currently do not know the precise molecular effects that cause formation of cells with no foci.

The fact that *oriC* activity is highest in triple-origin cells (Figure 2D) demonstrates that the capacity for *oriC* being active is highest in its native location, highlighting the importance of genome organization in the vicinity of *oriC*, and the importance of the location of *oriC* itself. We are only just beginning to appreciate the complexity of the three-dimensional structure of the nucleoid in bacterial cells. Indeed, changes of the *oriC* position were shown to alter the position of the Right and Left chromosomal macrodomains, highlighting that the position of *oriC* has a significant effect on chromosome organization (Duigou and Boccard, 2017). In addition, global gene order is surprisingly conserved between closely related prokaryotic species (Tamames, 2001). This order will get disrupted if additional origins are introduced into the chromosome, and we are only now starting to appreciate the effects this might have. Finally, the toxicity caused by oriY integration supports the idea that either the precise location of an active origin or the relative position of two active origins to each other can have strong effects (Dimude et al., 2018b), as observed (Kouzminova and Kuzminov, 2008).

## DNA Replication in Cells Without Active Replication Origins

The initiation of DNA synthesis at defined origins is a universal feature found in bacteriophages and viruses, prokaryotes, archaea, and eukaryotic cells (Costa et al., 2013). However, cells can survive without an active origin of replication. A recent study from the Allers lab (Hawkins et al., 2013) reported that *Haloferax volcanii*, a halophilic archaeon that grows in high salt environments under high osmotic pressure (Mullakhanbhai and Larsen, 1975), can not only tolerate deletion of all chromosomal origins, but grows with a doubling time faster than that of wild type cells (Hawkins et al., 2013). *Haloferax* cells contain a main chromosome, three secondary chromosomes, and a plasmid. High-resolution MFA revealed that the main chromosome is replicated from three origins, with a laboratory isolate showing a fourth, ori-pHV4, which is located in an integrated plasmid (Hawkins et al., 2013).

The deletion of single origins resulted in only mild growth rate reductions (Hawkins et al., 2013), as observed in other archaea (Samson et al., 2013; Wu et al., 2014). In contrast, deletion of multiple origins resulted in improved growth rates, and a derivative in which all replication origins were deleted grew faster than wild type cells, an effect that appears to be driven by recombination-dependent replication (Hawkins et al., 2013), replication that initiates at recombination intermediates (Hawkins et al., 2013; Michel and Bernander, 2014).

The ability to grow in the absence of replication origins is not a new finding. Kogoma and coworkers discovered that DNA intermediates involved in transcription (R-loops) and recombination (D-loops) can act as initiation points for DNA replication in *E. coli* (Kogoma and von Meyenburg, 1983). This type of synthesis was called constitutive stable DNA replication, or cSDR (Kogoma, 1997). DNA synthesis observed following DNA damage is a second type of stable DNA replication. This type requires induction of the SOS DNA damage response and was termed induced SDR (iSDR) (Kogoma, 1997).

Kogoma and co-workers described that cSDR in *E. coli* cells lacking RNase HI is persistent enough to allow successful cellular replication in the absence of an active *oriC* (Kogoma, 1997). It was suggested that the initiation at R-loops is the main driver of chromosome replication in these cells, because RNase HI specifically degrades RNA from DNA:RNA hybrids (de Massy et al., 1984; Kogoma, 1997; Tadokoro and Kanaya, 2009). In line with this idea, cSDR is also found in cells lacking the *topA* gene, which encodes for topoisomerase I. Topoisomerase I relaxes negative supercoiling to prevent the persistence of DNA-RNA hybrids. Consequently, cells lacking topoisomerase I show hyper-negative supercoiling, increased levels of R-loops, and cSDR (Brochu et al., 2018). R-loops can also arise when transcription fails to terminate. In *E. coli*, Rho-dependent transcription termination acts as a surveillance mechanism to keep pervasive transcription in check, which may otherwise lead to the formation of R-loops (Leela et al., 2013). Such R-loops may provide nucleating points for cSDR. Indeed, in strains mutated for *rho*, plasmids with a ColE1-like replication origin, which relies on R-loop formation for the initiation of synthesis, undergo runaway plasmid replication, and a combination of *rho* with other genes involved in R-loop removal caused synthetic lethality (Harinarayanan and Gowrishankar, 2003).

In recent studies, replication profiles revealed in more detail the locations where cSDR is initiated, which are reasonably well-defined, including one particularly strong site roughly 500–600 kb clockwise from *oriC* at ∼4.5 Mbp, as well as a peak of synthesis in the termination area (Maduike et al., 2014; Dimude et al., 2015; Veetil et al., 2020). Despite a detailed analysis of the locations of initiation sites, the precise molecular mechanism that triggers the initiation of DNA synthesis in these defined locations is not fully understood (Maduike et al., 2014; Dimude et al., 2015; Veetil et al., 2020). But the synthesis observed is strong enough to allow continuous replication of the entire chromosome in the absence of *oriC* firing, and cells lacking the *rnhA* gene, which encodes for RNase HI, can tolerate the deletion of the entire *oriC* area (Kogoma, 1997; Dimude et al., 2015). However, growth of ΔrnhA cells in the absence of *oriC* firing is slow and growth of dnaA(ts) ΔrnhA cells at restrictive temperature is sensitive to rich medium, such as LB broth (Kogoma, 1997). This broth-sensitivity can be partially alleviated by an rpoB^∗^35 allele (Dimude et al., 2015), a point mutation in the *rpoB* gene which encodes for the β subunit for RNA polymerase and which destabilizes ternary RNA polymerase complexes (Trautinger et al., 2005; Rudolph et al., 2007a). In addition, cells lacking RNase HI were reported to be synthetically lethal when also missing the homologous recombination proteins RecBCD (Itaya and Crouch, 1991), and this synthetic lethality can again be partially suppressed by an rpoB^∗^35 (called rpo^∗^ hereafter for simplicity) point mutation (Dimude et al., 2015), indicating that replication-transcription conflicts are a strong contributor to these effects.

RecBCD is involved in homologous recombination and is a key component needed for the processing of double-stranded DNA ends (Singleton et al., 2004). It binds to blunt or near-blunt double-stranded DNA (dsDNA) substrates (Dillingham and Kowalczykowski, 2008). RecB and RecD are both helicases, but they have different polarities: RecB is a 3′ to 5′ helicase, while RecD translocates in 5′ to 3′ direction (Dillingham and Kowalczykowski, 2008). Available dsDNA ends will be unwound and very rapidly degraded by the RecBCD complex (Dillingham and Kowalczykowski, 2008; Wiktor et al., 2018) until a chi site is reached (Smith, 2012). Chi sites are asymmetric octamers which can inhibit the degradation of the 3′ end by RecBCD while degradation of the 5′ end proceeds. Thus, upon reaching a chi site, degradation by RecBCD is modified so that a 3′ ssDNA overhang suitable for the loading of RecA recombinase is produced (Singleton et al., 2004).

It has become clear that RecBCD is very important for the resolution of intermediates that arise from replication-transcription conflicts (Syeda et al., 2016). RecBCD proved to be essential for the viability of fast-growing *E. coli* cells, in which one of the rrn operons was artificially inverted to force head-on replication-transcription encounters (De Septenville et al., 2012). The fact that Δ*oriC* ΔrnhA cells are broth sensitive and that ΔrecB ΔrnhA cells are synthetically lethal, with both effects being partially alleviated by an rpo^∗^ point mutation (Dimude et al., 2015), strongly suggests that DNA synthesis triggered at R-loops in cells lacking RNase HI in chromosomal areas away from *oriC* suffers from collisions with transcribing RNA polymerase complexes and requires processing by DNA repair and recombination proteins. Similarly, cells lacking Dam methylase, which has a role in strand-discrimination for methyl-directed mismatch repair, can grow in the absence of a functional origin, an effect that is likely to be caused by recombination-dependent replication triggered at now undirected MMR repair sites (Raghunathan et al., 2019). Analogously to cells lacking Rnase HI, an rpo^∗^ point mutation is one important factor that is required for Δdam cells to grow in the absence of *oriC* firing (Raghunathan et al., 2019).

## Replication Obstacles in Cells Carrying the Ectopic Replication Origin Oriz

While the deletion of all origins in *Haloferax* appears to allow faster growth of cells, at least under laboratory conditions (Hawkins et al., 2013), the same is not the case in bacteria such as *E. coli* and *B. subtilis*. Cells being forced to use initiation sites other than *oriC*, such as ΔrnhA cells in the absence of *oriC* firing, suffer considerable problems. Indeed, previous studies in *B. subtilis* where DNA replication initiated exclusively at an ectopic origin showed a substantial delay of replication at highly transcribed rrn operons encountered in an orientation opposite to normal (Wang et al., 2007; Srivatsan et al., 2010).

In Δ*oriC* oriZ^+^ cells the chromosome is replicated exclusively from the ectopic oriZ, very similar to the described situation in *B. subtilis*. It was therefore a surprise when Wang et al. (2011) reported that Δ*oriC* oriZ^+^ cells grew with a doubling time very similar to that of wild type cells. Indeed, when we re-generated a Δ*oriC* oriZ^+^ construct, we found its doubling time to be over 40 min. Δ*oriC* oriZ^+^ cells seriously struggle to grow and rapidly accumulate suppressor mutations that allow faster growth (Ivanova et al., 2015).

**FIGURE 3:**
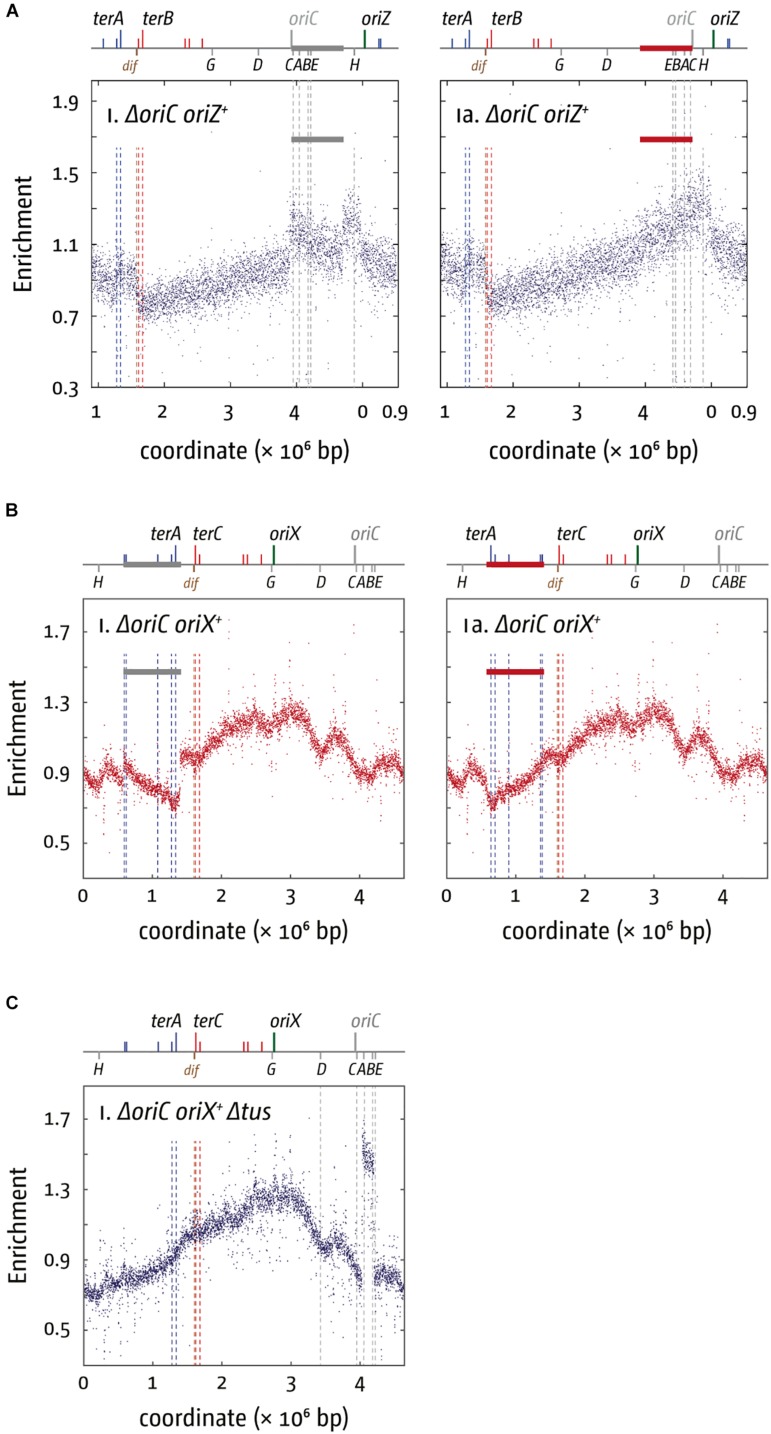
Chromosomal rearrangements in *E. coli* cells replicating from a single ectopic replication origin. **(A)** Replication profiles of *E. coli* cells with a single ectopic replication origin. Shown is the marker frequency analysis of *E. coli*Δ*oriC* oriZ^+^ cells. The number of reads (normalized against reads for a stationary phase wild type control) is plotted against the chromosomal location. A schematic representation of the *E. coli* chromosome showing positions of *oriC* (gray to indicate the deletion) and oriZ (green line) and ter sites (above) as well as dif and rrn operons A–E, G, and H (below) is shown above the plotted data. A clear discontinuity of the profile can be seen in **(panel i)** (marked by a gray bar), which is due to a large inversion, as highlighted by the continuous replication profile that results if the area highlighted (red bar indicates the inverted area) is inverted. The MFA raw data were taken from Ivanova et al. (2015) and re-plotted to allow changes the scale of the plots, if necessary, and to highlight specific schematic features of the *E. coli* chromosome. **(B)** Replication profiles of *E. coli*Δ*oriC* oriX^+^ cells. A clear discontinuity of the profile can be seen in **panel i** (marked by a gray bar), which is due to a large inversion, as highlighted by the continuous replication profile that results if the area highlighted (red bar indicates the inverted area) is inverted. The MFA raw data were taken from Dimude et al. (2018b) and re-plotted to allow changes the scale of the plots, if necessary, and to highlight specific schematic features of the *E. coli* chromosome. **(C)** Replication profiles of *E. coli*Δ*oriC* oriX^+^ Δtus cells. A clear discontinuity of the replication profile can be seen between the rrn operons A and B, which is due to a duplication of the entire region. See text for details.

The replication profile of Δ*oriC* oriZ^+^ cells revealed two major obstacles to replication. The asymmetry of the replichore arrangement is even more extreme in Δ*oriC* oriZ^+^ cells than in *oriC*^+^ oriZ^+^ cells, as the fork traversing counterclockwise has to replicate 3/4 of the entire chromosome. Consequently, the “step” within *terA* and *terC* is strongly pronounced (Ivanova et al., 2015). But replication initiated at oriZ and traversing the chromosome counter-clockwise also encounters the highly transcribed rrnH and rrnCABE operons in an orientation opposite to normal (Figure 2A), resulting in significant problems (Ivanova et al., 2015), in line with results in *B. subtilis* (Wang et al., 2007, 200; Srivatsan et al., 2010). A clear prediction of these observations is that the slow growth phenotype of Δ*oriC* oriZ^+^ cells should be suppressed by two classes of mutations: the inactivation of the replication fork trap as well as any mutation that causes a reduction of the severity of conflicts between replication and transcription. This is indeed what we observed. The slow growth phenotype of Δ*oriC* oriZ^+^ cells was partially suppressed by the inactivation of the replication fork trap (Δtus) and an rpo^∗^ point mutation (Ivanova et al., 2015). However, the fast growth of the original Δ*oriC* oriZ^+^ construct by Wang et al. (2011) was caused by a suppressor mutation that solved the problem in a far more elegant way: their fast growing Δ*oriC* oriZ^+^ strain carried a substantial inversion. This inversion spanned, with the exception of rrnH, almost the entire remaining portion of the chromosome that would have been replicated in the wrong orientation from oriZ, including the entire rrnCABE operon cluster. Thus, the problem in these cells was solved simply by the re-alignment of replication and transcription (Figure 3A; Ivanova et al., 2015), strongly supporting to the notion that avoiding head-on collisions has significantly contributed to shaping the distinct architecture of bacterial chromosomes.

This idea is further supported by the fact that a variety of different repair systems are present in cells dedicated to dealing with tightly bound DNA-protein complexes. In *E. coli*, a variety of helicases promote fork progression through tightly bound nucleoprotein complexes, including Rep, UvrD, and DinG (Guy et al., 2009; Boubakri et al., 2010; Atkinson et al., 2011). Rep is considered an accessory replicative helicase because Rep physically associates with the replicative helicase, DnaB (Bochman et al., 2010; Brüning et al., 2014; Syeda et al., 2019). Chromosome duplication takes almost twice as long in Δrep cells than in wild type cells (Lane and Denhardt, 1975; Guy et al., 2009; Atkinson et al., 2011). In addition, enzymes involved in homologous recombination play an important role in assuring that replication forks move successfully through highly transcribed areas (Cox, 2001; Dillingham and Kowalczykowski, 2008; Boubakri et al., 2010; De Septenville et al., 2012; Michel et al., 2018), as already discussed above.

**FIGURE 4:**
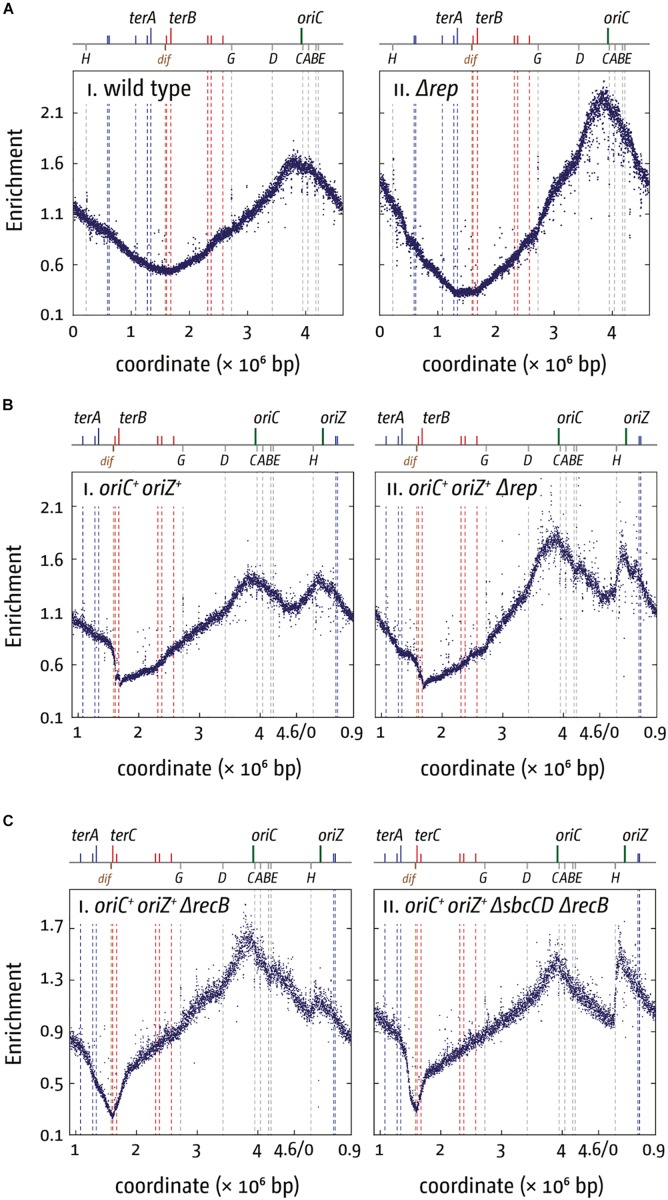
Replication dynamics and cell viability in cells with one or two active replication origins lacking either Rep helicase or RecBCD exonuclease. **(A)** Cells lacking Rep helicase show an increased origin/terminus ratio than wild type cells, indicating that replication fork progression is significantly slowed. The replication profiles are generated by plotting the number of sequence reads (normalized against reads for a stationary phase wild type control) against their chromosomal location. The schematic representation of the *E. coli* chromosome above each panel shows the positions of the two origins, *oriC* and oriZ, and ter sites (above) as well as the dif chromosome dimer resolution site and rrn operons A–E, G, and H (below). **(B)** Replication fork progression is blocked at the highly transcribed rrnH operon replicated in a direction opposite to normal in *oriC*^+^ oriZ^+^ cells lacking Rep helicase. Please note that the chromosomal coordinates are shifted in comparison to panel **(A)** so that *oriC* and oriZ next to each other. **(C)** Replication fork progression is arrested at rrnH if replication proceeds in an orientation opposite to normal, and oriZ peak height is much reduced in cells lacking RecBCD exonuclease **(panel i)**. oriZ peak height is restored if SbcCD is missing in addition to RecBCD **(panel ii)**. See text for details. For an in-depth discussion of the underrepresentation of sequence reads in the termination area please refer to Wendel et al. (2014), Sinha et al. (2017, 2018), and Dimude et al. (2018a). All raw data in panels **(A–C)** are taken from Dimude et al. (2018a) and re-plotted to allow changes the scale of the plots, if necessary, and to highlight specific schematic features of the *E. coli* chromosome. As for panel **(B)**, please note that the chromosomal coordinates for panel **(C)** are shifted in comparison to panel **(A)** so that *oriC* and oriZ are next to each other.

Why are a variety of repair systems needed to deal with stably bound DNA-protein complexes? When we investigated viability and replication profiles of cells lacking Rep helicase, we found a much-increased origin/terminus ratio (cf. Figures 4Ai,ii; Dimude et al., 2018a). The replication profiles reveal no specific areas that appear problematic. As replication profiles are population-based, this observation suggests that Rep acts on average at sites relatively evenly distributed throughout the chromosome. However, the replication profile of *oriC*^+^ oriZ^+^ Δrep cells revealed that the progression of DNA replication is very effectively blocked by rrn operons encountered in a head-on orientation, as indicated by the rather abrupt change of the replication gradient at rrnH (cf. Figures 4Bi,ii). Indeed, Δ*oriC* oriZ^+^ Δrep cells are inviable (Dimude et al., 2018a). Viability is restored by an rpo^∗^ mutation in which replication-transcription conflicts are lessened, and the replication profiles show that synthesis can indeed proceed (Dimude et al., 2018a).

rrn operons encountered in a head-on orientation in cells lacking RecBCD block replication even more severely than in cells lacking Rep, and there is no indication of replisomes proceeding past rrnH, the first rrn operon encountered (Figure 4Ci). Δ*oriC* oriZ^+^ ΔrecB cells are inviable unless an rpo^∗^ point mutation is present, but even then, cells can only survive in minimal medium, in which a reduced growth rate means a slower doubling time and a reduced demand for rRNA in comparison to growth in rich medium. They remain synthetically lethal in LB and our replication profiles show that replication proceeds past rrnH with a low frequency, low speed, or both (Dimude et al., 2018a).

Replication profiles of *oriC*^+^ oriZ^+^ ΔrecB cells also showed a much-reduced peak height of oriZ, while firing of *oriC* appeared to be unaffected (Figure 4Ci). Peak height was restored in cells also lacking the exonuclease SbcCD (Figure 4Cii), indicating that extensive SbcCD-dependent degradation takes place in the absence of RecBCD at replication forks arrested at highly transcribed rrn operons (Dimude et al., 2018a).

Taken together, the data currently available suggest that replication-transcription conflicts can trigger different type of arrested forks, depending, for example, on the level of transcription. Indeed, it was shown that the mode of protein displacement of nucleoprotein complexes by RecBCD helicase/exonuclease varies depending on overall protein density (Terakawa et al., 2017). The different types of arrested of perhaps even collapsed replisomes then will require different types of processing that have to take place (2016; Dimude et al., 2018a). Replication coming from oriZ will encounter several genes that are transcribed in an orientation opposite to normal, and both co-directional as well as head-on conflicts are problematic (Merrikh et al., 2011, 2012; Lang and Merrikh, 2018). Nevertheless, there is no indication of any substantial block to replication in ΔrecB cells until the first highly transcribed region is reached. It appears that Rep helicase is sufficient to facilitate replisome progression through these areas. But when forks encounter a rrn operon in an orientation opposite to normal the situation differs significantly. The intermediates generated in this situation appear to be accessible to degradation by SbcCD (Dimude et al., 2018a) and other nucleases such as RecJ (De Septenville et al., 2012) and are extensively resected. In addition, ΔuvrD Δrep rpo^∗^ cells can only survive in the presence of both RecBCD and RecA, suggesting that the loading of RecA by RecBCD is required for the continuation of DNA replication (Syeda et al., 2016). Thus, both the failing to load RecA and the extensive resection contribute to *rrnH* being such a severe block in *oriC*^+^ oriZ^+^ ΔrecB cells (Dimude et al., 2018a). In contrast, no obvious resection is observed in cells lacking Rep (Dimude et al., 2018a).

It was suggested that DinG is an additional protein that is involved in aiding the progression of replication through highly transcribed areas of the chromosome (Baharoglu et al., 2010; Boubakri et al., 2010). Indeed, we were able to show that Δ*oriC* oriZ^+^ ΔdinG cells are synthetically lethal, an effect robustly suppressed by a rpo^∗^ point mutation. This result supports the idea that DinG is involved in underpinning replication of highly transcribed areas in *E. coli*. However, DinG is unable to directly promote replisome movement through stalled transcription complexes *in vitro*, and the replication profile of *oriC*^+^ oriZ^+^ ΔdinG cells do not reveal any abnormalities at rrnH (Hawkins et al., 2019), much in contrast to cells lacking either Rep or RecB (Dimude et al., 2018a). Thus, it seems that DinG might have an indirect effect in resolving replication-transcription encounters, potentially via its ability to unwind RNA:DNA hybrids (Voloshin and Camerini-Otero, 2007).

## Replication Obstacles in Cells Carrying the Ectopic Replication Origin Orix

The results described so far strongly support the idea that replication-transcription conflicts are an important factor that have contributed to shaping the structure of bacterial chromosomes. In line with this idea, replication-transcription conflicts came up again when we tried to generate Δ*oriC* oriX^+^ cells. One rationale of integrating an ectopic replication origin into the left-hand replichore in the first place was the fact that there is only a single rrn operon (rrnD) between the integration location and *oriC*, together with a cluster of highly transcribed genes that code for ribosomal proteins. Thus, we speculated that replication-transcription conflicts might be less severe in this particular construct, whereas replication in Δ*oriC* oriZ^+^ cells has to overcome 5 highly transcribed rrn operons. However, the fact that the rpo^∗^ mutation improved doubling times of various Δ*oriC* oriX^+^ constructs suggests that conflicts still have a considerable impact (Dimude et al., 2018b).

Our studies in oriX cells revealed that, beside replication-transcription conflicts, the replication fork trap severely impacts on genome duplication in *oriC*^+^ oriX^+^ and Δ*oriC* oriX^+^ cells. In fact, similar to the situation in Δ*oriC* oriZ^+^ cells initially described (Wang et al., 2011; Ivanova et al., 2015), we found that our Δ*oriC* oriX^+^ construct contained a large inversion. This inversion spanned all blocking ter sites and flipped them into permissive orientation. Thus, the inversion allows replication to proceed unhindered (Figure 3B), demonstrating the impact of the replication fork trap on replication progression (Dimude et al., 2018b). The inversion also re-aligns the direction of replication and transcription in the way it is in *oriC* cells, and both the replication fork trap and replication-transcription conflicts might be an important factor here. However, if transcription generally interferes with replication, a prediction is that for both *oriC*^+^ oriX^+^ Δtus and *oriC*^+^ oriZ^+^ Δtus cells forks escaping the termination area should be slowed down, as their progression into the opposite replichore would force an increased number of head-on collisions. If forks escaping the termination area are slower than forks coming from the native *oriC*, the fork fusion point should be shifted from the location equidistant to both origins toward the termination area. However, this is not what we observed. In *oriC*^+^ oriX^+^ Δtus cells the fork fusion point was close to the arithmetic mid-point between *oriC* and oriX and only slightly shifted toward the termination area (∼20 kb) (Dimude et al., 2018b), while for *oriC*^+^ oriZ^+^ Δtus cells forks terminated 60 kb in the direction of *oriC* (Ivanova et al., 2015; Dimude et al., 2016). We do not have any direct information about the speed of individual forks, but these results suggest that the forks leaving the termination area and traveling in the wrong orientation have, on average, a similar speed to the forks coming from *oriC* (oriX) or are even slightly faster (oriZ) (Ivanova et al., 2015; Dimude et al., 2016, 2018b), similar to the situation observed in *Vibrio cholerae* where replication forks simply fused opposite the origin even when the origin was moved to an ectopic location (Galli et al., 2019).

A clue for an additional factor that might contribute to replication dynamics and genome structure comes from the observation that one of our Δ*oriC* oriX^+^ Δtus constructs had acquired a spontaneous duplication of the chromosomal stretch containing rrn operons *A* and *B* (Figure 3C). Highly transcribed genes tend to be located in relative vicinity to the origin. In fast growing cells this area can be in a ratio of four to one relative to the termination area or even higher. The increased number of gene copies results in a gene dosage effect (Jin et al., 2012). The rrn operons *CABE* and *D* are all located in close proximity to *oriC*, causing an increased gene dosage in fast-growing cells (Jin et al., 2012). If, however, the origin is shifted from its original location into the left-hand replichore, rrn operons CABE and H are all in quite a distance from the active origin, which results in a lower copy number. This effect will be less pronounced in Δ*oriC* oriZ^+^ cells, because the location of oriZ is in close proximity to rrnH and the rrnCABE cluster. It was reported before that inactivation of up to three of the rrn operons in *E. coli* caused significant upregulation of the remaining rrn operons, thereby compensating for the reduced copy number (Condon et al., 1993). However, especially if multiple rrn operons are affected, a reduced growth rate was observed (Condon et al., 1993).

Chromosomal replication starting exclusively at oriX will transfer especially the rrnCABE cluster and rrnH into a completely different chromosomal environment, as movement of the *oriC* position was shown to alter the position of the chromosomal macrodomains (Duigou and Boccard, 2017). Indeed, it was shown that expression of a reporter cassette under control of the lac promoter showed a 300-fold variation in transcription levels depending on its precise integration location into the chromosome (Bryant et al., 2014; Scholz et al., 2019), and displacement of pleiotropic genes were indeed shown to affect the phenotype and competitive growth fitness of cells (Gerganova et al., 2015). If rrnCABE and H are less transcribed in Δ*oriC* oriX^+^ cells this might contribute to the explanation why we struggled particularly with the generation of Δ*oriC* oriX^+^ cells, and it would suggest that the observed duplication of rrnA and B are indeed beneficial to the competitive fitness of our Δ*oriC* oriX^+^ Δtus construct (Dimude et al., 2018b). It might also explain why the inversion found in the initial Δ*oriC* oriZ^+^ construct generated in the Sherratt Lab (Wang et al., 2011) is a particularly efficient suppressor, as it not only realigns replication and transcription, but also brings the rrnCABE cluster back into close proximity of the only active origin, oriZ (Ivanova et al., 2015).

## Making Sense of the Replication Fork Trap

The gross chromosomal rearrangement in Δ*oriC* oriX^+^ cells that flipped all ter sites from blocking into permissive orientation strongly highlights the constraint imposed by such a replication fork trap on genome duplication (Dimude et al., 2018b). Any arrest of one of the two forks cannot be alleviated by simply waiting until the second fork arrives, as this fork will be blocked by the fork trap. However, ter/Tus complexes are not systematically involved when replication forks fuse. Early labeling experiments (Bouché et al., 1982), and more recently MFA (Rudolph et al., 2013; Ivanova et al., 2015; Dimude et al., 2016), indicates that in wild type *E. coli* cells the majority of forks fuse close to the arithmetic mid-point, somewhere between the dif chromosome dimer resolution site and *terC* (Rudolph et al., 2013; Ivanova et al., 2015; Dimude et al., 2016). Thus on a population basis both the clockwise and counterclockwise fork appear to move normally with similar speeds, which results in a fusion of two freely moving replisomes within the innermost ter sites, at least under laboratory conditions. It seems that the fork trap mostly comes into play upon a delay of one of the two forks at an obstacle, such as a nucleoprotein complex or a DNA lesion.

If the replication fork trap is not systematically involved in termination what might be its physiological role? Together with a single origin of replication, it certainly contributes to strictly maintaining replicational directionality within the two replichores, and it was suggested that a fork trap is important to maintain the co-directionality of transcription and replication (Brewer, 1988; Rudolph et al., 2007a). Given the strong impact of replication-transcription clashes described above and elsewhere, and the many repair pathways dealing with such conflicts, this will be an important factor (McGlynn et al., 2012; Merrikh et al., 2012; Lang and Merrikh, 2018).

However, it appears that many bacterial species do not utilize a dedicated fork trap (Galli et al., 2019). And, in *E. coli*, genome-wide co-directionality of replication and transcription is only approximately 55% (McLean et al., 1998). The vast majority of highly transcribed genes are transcribed co-directionally with replication (McLean et al., 1998), but all rrn operons and the majority of genes encoding for ribosomal proteins are in relative proximity to the origin (Jin et al., 2012; Dimude et al., 2016). Thus, a fork escaping the fork trap in *E. coli* would have to proceed for about 1 Mbp (1/4 of the chromosome) before any of the genes transcribed at very high levels would be reached (Figure 1A). Indeed, as highlighted above, replication in the vicinity of the termination area appears to proceed with speeds similar to replication coming from *oriC*, both in the left- and right-hand replichore (Ivanova et al., 2015; Dimude et al., 2018b). This observation does not rule out replication-transcription conflicts, as forks coming from *oriC* might also suffer from delays and we did not directly measure fork speed. However, for tRNA genes, which are highly transcribed under fast growth conditions and which are more globally distributed throughout the chromosome, we found a co-directionality of replication and transcription in the origin-proximal half of the chromosome only. In the origin-distal half relative orientation of replication and transcription is much more variable. Indeed, we were surprised to find a mild bias toward the head-on orientation for replication coming from *oriC* (Dimude et al., 2016). Thus, while avoiding clashes between replication and transcription is important, it remains debatable whether avoiding such clashes is the main purpose of the fork trap in *E. coli*.

Is the absence of the replication fork trap causing any phenotypes which might shed light on its physiological role? When working with oriX^+^ and oriZ^+^ strains we noticed that deletion of tus consistently caused a mild growth defect (Ivanova et al., 2015; Dimude et al., 2018b). This suggests that restricting fork movement in the termination area somehow facilitates replication completion or successful chromosome segregation or both.

One process that is uniquely happening in the termination area is the fusion of the two replication forks. Could this process itself, or some unwanted side effect, be responsible for the observed delay? Various experimental approaches have shown that an absence of functional ter/Tus complexes can result in replication still occurring when it is meant to stop. Because replication continues to occur when a complete copy of the DNA is generated, we call this continued synthesis over-replication, as it over-replicates molecules that are already fully replicated. This was observed for plasmid R1 in *E. coli*. R1 is replicated unidirectionally by a single fork until it gets arrested at a single ter/Tus complex close to the plasmid origin (Nordström, 2006). Inactivation of this stopping point for replication allows synthesis to proceed into an already replicated area, and this was shown to result in the accumulation of branched DNA structures, rolling circle replication intermediates and the formation of plasmid multimers (Krabbe et al., 1997). It was suggested that, upon reaching an already replicated area, the replicative helicase of the fork might displace already existing nascent strands. The resulting intermediates can then serve as substrates at which additional synthesis can proceed (Krabbe et al., 1997). Similarly, it was shown *in vitro* that for a plasmid substrate functional ter/Tus complexes efficiently prevented over-replication and the formation of complex intermediates (Hiasa and Marians, 1994), a result that was recently confirmed in a reaction using the elegant “replication chain reaction” (Hasebe et al., 2018). These results indicate that the fork trap can prevent unwanted over-replication that is linked to termination of DNA synthesis.

Similarly, it was found that Δtus cells showed chromosomal over-replication, even though at a low level (Markovitz, 2005). This effect was exacerbated by point mutations in DNA polymerase I (Markovitz, 2005), which has a prominent role in the repair of DNA damage and the maturation of Okazaki fragments (Kurth and O’Donnell, 2009), leading to the suggestion that Pol I might be involved in bringing DNA replication to a successful conclusion in the terminus region (Markovitz, 2005). Results from *B. subtilis* suggest that the absence of the Rtp terminator protein can result in the formation of an increased number of chromosomal dimers (Lemon et al., 2001; Duggin et al., 2008). Since over-replication results in the generation of double-stranded DNA ends accessible to homologous recombination (Figure 6 and below), the increased formation of chromosome dimers could be a result of problems with fusing replisomes, similar to the situation in *E. coli*.

**FIGURE 5:**
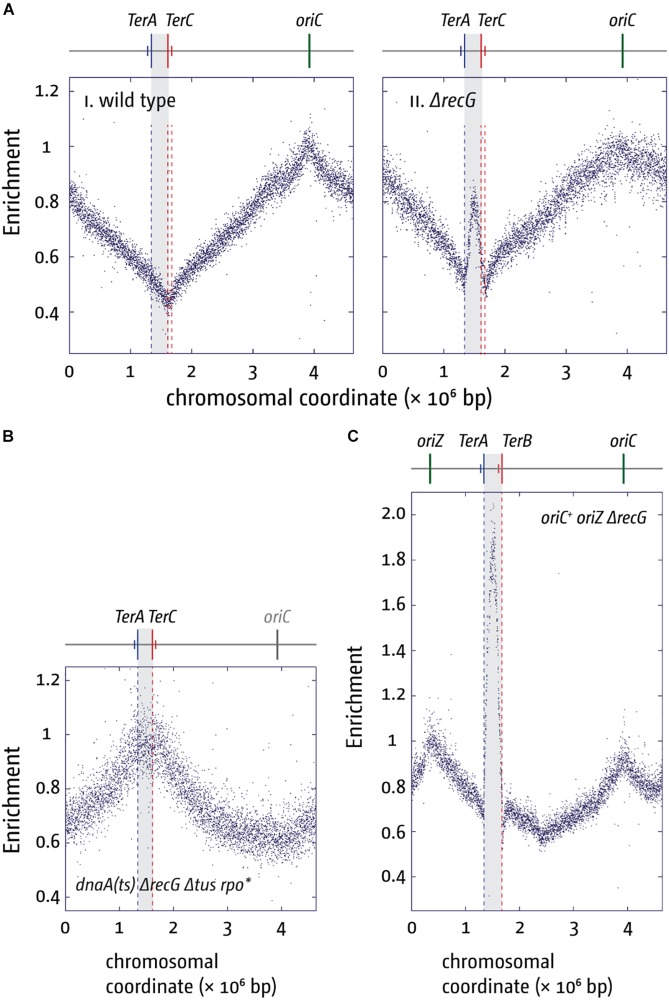
Over-replication in the termination area in the absence of RecG helicase. **(A)** Replication profiles of *E. coli* cells in exponential phase. Cells were grown at 37°C. The number of reads (normalized against the reads for a stationary wild type control) is plotted against the chromosomal coordinate. Positions of *oriC* (green line) and primary ter sites are shown above the plotted data with red and blue lines representing the left and right replichore, as depicted in Figure 1A. The termination area between the innermost ter sites is highlighted in light gray. **(B)** Marker frequency analysis of a ΔrecG Δtus rpo* strain that carries a temperature-sensitive allele of the main replication initiator protein DnaA. The strain was grown at 42°C to inactivate DnaA(ts) and therefore prevent *oriC* firing. **(C)** Marker frequency analysis of chromosome replication in *oriC*^+^ oriZ^+^ strain in the absence of RecG. Strains were grown at 37°C. The raw data in panels **(A–C)** were taken from Rudolph et al. (2013) and re-plotted to allow changes the scale of the plots, if necessary, and to highlight specific schematic features of the *E. coli* chromosome.

An even stronger effect of a Δtus mutation was found in cells lacking RecG helicase. The replication profile of ΔrecG cells shows a peak of over-replication within the four innermost ter sites (cf. Figures 5Ai,ii; Rudolph et al., 2013; Wendel et al., 2014; Dimude et al., 2015, 2016; Midgley-Smith et al., 2018b). Indeed, this over-replication can support growth in the absence of a functional origin if a) a functional replication fork trap is absent (Δtus) and b) replication-transcription conflicts resulting from forks leaving the termination area and proceeding in an orientation opposite to normal are alleviated (rpo^∗^) (Rudolph et al., 2013). In the absence of *oriC* activity ΔrecG Δtus rpo^∗^ cells show a replication profile that is inverted: the *oriC* area shows a low-point of the profile while, rather paradoxically, the highest point of the profile is observed in the termination area where forks normally fuse to end DNA synthesis (Figure 5B; Rudolph et al., 2013; Dimude et al., 2015).

**FIGURE 6:**
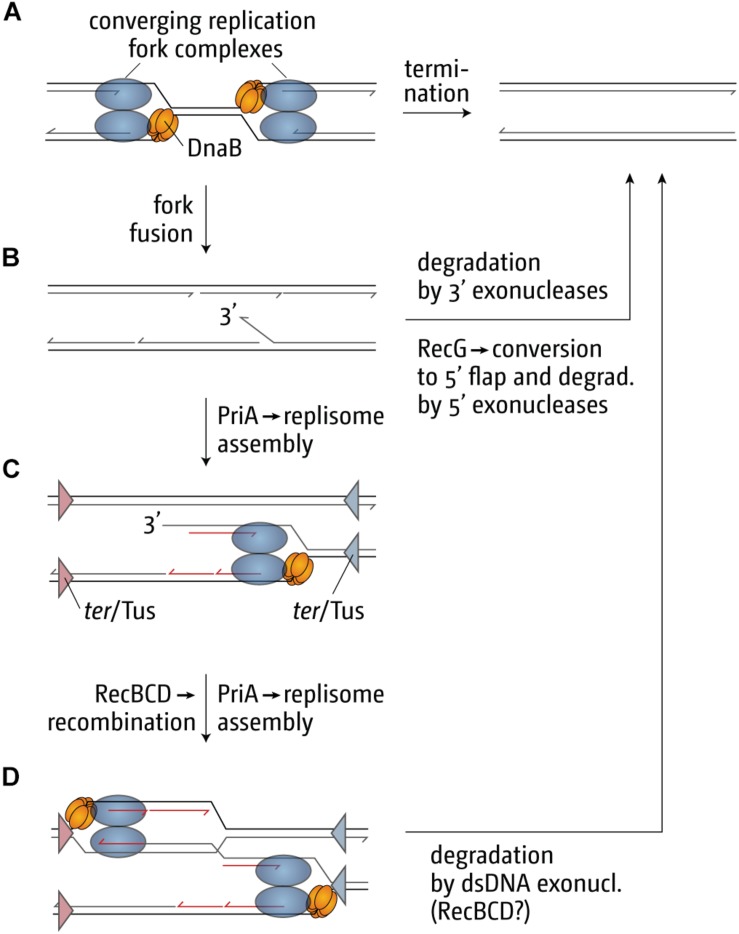
Illustration of how replication fork fusions might trigger over-replication in the termination area and how this is normally prevented by proteins such as RecG and/or 3′ exonucleases. The fusion of two replisomes **(A)** can result in the formation of key intermediates, such as a 3′ single-stranded DNA flap **(B)**, which can be processed by restart proteins such as PriA **(C,D)** if it is not removed or degraded. ter/Tus complexes are shown in panels **(C,D)** as triangles. The blue ter/Tus complexes are oriented such that they would block synthesis initiated within the termination area and moving counterclockwise, while the red ter/Tus complexes would block clockwise synthesis. As these complexes are permissive for the forks coming from *oriC* in panel **(A)** they have been excluded for simplicity. Note that, while the formation of a 3′ flap can occur at both forks, only one such reaction was shown for simplicity. See text for details.

Our genetic analysis of the over-replication in ΔrecG cells suggests that it is triggered by intermediates which are similar to those proposed for replication of plasmid R1 (Krabbe et al., 1997; Rudolph et al., 2013; Dimude et al., 2015, 2016; Lloyd and Rudolph, 2016; Midgley-Smith et al., 2018b). We believe that upon fusion of two replication forks an intermediate is generated that allows either the continuation of synthesis or the re-recruitment of new forks. The over-replication in ΔrecG cells strictly requires the ability of the main restart protein PriA to process a 3′ flap structure (Rudolph et al., 2013). In addition, we observed that over-replication also occurs in cells lacking 3′ exonucleases Exo I, Exo VII, and SbcCD (Rudolph et al., 2010a, 2013; Midgley-Smith et al., 2018a). These results indicate that a 3′ flap might be a central intermediate. We have proposed that such a 3′ flap might arise upon the fusion of two forks by the displacement of the nascent leading strand of one of the two forks by the replicative helicase of the other (Figure 6B). 3′ flaps were shown to be a very good substrate for RecG helicase *in vitro* (McGlynn and Lloyd, 2001; Tanaka and Masai, 2006; Rudolph et al., 2010b; Bianco, 2015) and, in its presence, would be rapidly converted into 5′ flaps or, alternatively, degraded by 3′ exonucleases (Figure 6B; Rudolph et al., 2013; Dimude et al., 2016; Midgley-Smith et al., 2018a). If a 3′ flap remains unprocessed, PriA might gain access and re-recruit a replisome (Figure 6C), leading to the observed over-replication of the termination area. However, such newly initiated synthesis would generate double-stranded DNA ends (Figure 6C). dsDNA ends will be rapidly processed by RecBCD and RecA, resulting in the formation of a D-loop (Rudolph et al., 2009, 2010a, 2013; Dimude et al., 2016), another substrate at which PriA can establish a functional replisome (Figure 6D). Progression of forks established in this way will proceed until they get blocked at a ter/Tus complex (Figure 6D).

Rather than by fork fusion events themselves, might the over-replication be caused by a cryptic origin that is normally suppressed, or by the increased occurrence of R-loops within the termination area, as recently suggested (Kuzminov, 2016)? While remaining a possibility, it is unlikely for a number of reasons. Firstly, we observed that linearization of the chromosome within the termination area much reduced the over-replication both in ΔrecG cells and in cells lacking 3′ exonucleases (Rudolph et al., 2013; Dimude et al., 2015; Midgley-Smith et al., 2018a). While linearization would prevent two replisomes from fusing, it will not interfere with the activity of a cryptic origin, and we have indeed observed that linearization of the chromosome in cells lacking RNase HI does not abolish the R-loop-driven over-replication in the termination area (Dimude et al., 2015). Secondly, we observed that over-replication in the termination area is dramatically exacerbated in ΔrecG cells if oriZ is introduced (Figure 5C; Rudolph et al., 2013; Midgley-Smith et al., 2018b). It is not clear how integration of an origin ∼1 Mbp away from the termination area should cause such a dramatic increase in activity of either a cryptic origin or a hot spot for R-loop formation, while it clearly changes fork fusion events (Midgley-Smith et al., 2018a, b). Thirdly, we were recently able to demonstrate that over-replication can indeed be triggered outside of the termination area in *oriC*^+^ oriZ^+^ ΔrecG cells. In *oriC*^+^ oriZ^+^ cells, a second fork fusion event takes place in an ectopic location, and we were able to show that this ectopic fork fusion event can also trigger over-replication (Midgley-Smith et al., 2018b). Thus the over-replication is not location-bound but can be observed in other chromosomal contexts if forks are forced to fuse in this area. In addition, it is also not clear how proteins such as 3′ exonucleases and DNA polymerase I, would be involved in suppressing a cryptic origin or R-loops (Markovitz, 2005; Rudolph et al., 2013; Midgley-Smith et al., 2018a, b). Taken together, we prefer the idea that fork fusion intermediates are responsible for triggering the over-replication observed, as it fits the available data much better than a cryptic origin or a R-loop hotspot.

If so, might the fork trap provide a defined chromosomal region where termination intermediates and the resulting over-replication can be contained and quickly and safely processed to bring DNA replication to an accurate conclusion? The termination area was found to be a recombination hotspot (Horiuchi et al., 1994), a result that would be easily explained if over-replication intermediates, that can arise occasionally despite the presence of all processing factors in wild type cells, would trigger increased levels of recombination (Rudolph et al., 2013; Dimude et al., 2016; Midgley-Smith et al., 2018a, b). Increased recombination frequencies as well as chromosomal over-replication contribute significantly to genomic instability (Finkel et al., 2007; Blow and Gillespie, 2008; Alexander and Orr-Weaver, 2016; Tomasetti et al., 2017), highlighting why a fork trap might be beneficial. However, the relatively mild phenotype of cells lacking a fork trap system highlights that this effect is in addition to the various processing factors that are involved in the processing of fork fusion intermediates, such as RecG, 3′ exonucleases and DNA polymerase I. These proteins seem to be able to deal with occurring intermediates efficiently, which might explain why a fork trap system is found in only a limited number of bacterial species (Galli et al., 2019). Nevertheless, in species in which the opportunity arose (Galli et al., 2019), the fork trap system might have been a welcome addition, and as highlighted before, the effect on the doubling time, although small, is measurable (Ivanova et al., 2015; Dimude et al., 2018b).

The importance of the proteins dealing with fork fusion intermediates is highlighted by the synthetic lethality of cells that lack combinations of the proteins involved. Cells lacking both RecG and 3′ exonucleases are synthetically lethal (Rudolph et al., 2010a), as are cells lacking both RecG and DNA polymerase I (Hong et al., 1995; Zhang et al., 2010; Upton et al., 2014). Furthermore, we found that Δ*oriC* oriZ^+^ ΔrecG cells are synthetically lethal, an effect that is suppressed by the inactivation of the replication fork trap, suggesting that the lethality is caused by the vastly exacerbated levels of over-replication in the termination area (Midgley-Smith et al., 2018b). However, over-replication is not triggered by forks arrested at ter/Tus complexes, as it is still observed in ΔrecG cells lacking Tus terminator protein (Rudolph et al., 2013; Midgley-Smith et al., 2018b).

## Concluding Remarks

While genome sizes and certain structural aspects of bacterial genomes show considerable variability, all bacterial chromosomes investigated so far have in common that they are duplicated by two replication forks initiated at a single origin (Gao and Zhang, 2008; Gao, 2015). Additional active origins can be introduced into the chromosome, but in the existing chromosome structure they always cause a disadvantage, such as a mild growth defect (oriX, oriZ) (Ivanova et al., 2015; Dimude et al., 2018b), silencing of one of the active origins (Kouzminova and Kuzminov, 2008) or causing some sort of toxicity to cells (oriY) (Dimude et al., 2018b). The observed problems are, at least in part, caused by genome trafficking problems, such as conflicts between replication and transcription, tightly bound protein-DNA complexes such as ter/Tus complexes, and other related issues, highlighting these processes as likely contributors of the overall structure of bacterial chromosomes, as suggested in a variety of other studies. Indeed, the finding that replication and transcription are aligned in human cells as well via the positioning of origins relative to highly transcribed genes (Chen et al., 2019) suggests that this is a very universal feature of nucleic acid metabolism. However, while the strict replichore arrangement in bacteria allows for an easy way to co-align replication and transcription, the results in human cells demonstrate that this can also be achieved in more complex environment where hundreds of origins are active.

Another process that might have contributed to shaping the landscape of bacterial chromosomes is the fusion of two converging replication forks. Work done by our lab as well as others has identified a surprising number of proteins that are involved in preventing over-replication in the termination area (Krabbe et al., 1997; Markovitz, 2005; Rudolph et al., 2013; Wendel et al., 2014, 2018; Midgley-Smith et al., 2018a, b), and we suggest that this is large number is needed for the processing of intermediates that arise directly as a result of forks fusions (Rudolph et al., 2009, 2010a; Dimude et al., 2016; Lloyd and Rudolph, 2016; Figure 6). Indeed, the lethality observed when multiple of these processing activities are removed from cells (Hong et al., 1995; Rudolph et al., 2010a; Zhang et al., 2010; Upton et al., 2014; Midgley-Smith et al., 2018b) highlights the importance of dealing with such intermediates. If the fusion of two forks can have harmful consequences, one easy way to limit these events is simply by reducing the number of origins. Having precisely one origin allows not only the easy co-orientation of highly transcribed genes with DNA synthesis, but also reduces the number of fork fusion events to exactly one under normal conditions. Proteins and the replication fork trap allow then for the quick and efficient processing of potentially harmful fork intermediates (Dimude et al., 2016; Midgley-Smith et al., 2018a, b). The mild phenotypes of cells lacking a fork trap suggests that the various proteins involved can deal with fork fusion intermediates quite efficiently. Thus, acquiring the fork trap from a plasmid (Galli et al., 2019) might have been a welcome additional help to deal with these events, but it is not essential, explaining perhaps why many other bacterial species do not utilize a fork trap mechanism.

This hypothesis might help to explain why a transition from strictly single to both single and multiple origins took place in archaea. In both archaea and eukaryotic cells, the replicative helicase has the opposite polarity to the replicative helicase in bacteria (Tuteja and Tuteja, 2004; Costa and Onesti, 2008, 2009; Sakakibara et al., 2009) and encircles the single stranded leading strand template (Bai et al., 2017). Okazaki fragments in eukaryotes are much shorter than in prokaryotes (Burgers, 2009), allowing the replicative helicase to simply unwind either one or perhaps even more un-ligated Okazaki fragments. But even if any strand displacement would occur upon the merging of two forks, this would result in the generation of a 5′ flap which would be processed by a 5′ nuclease, such as the flap endo nuclease FEN-1 (Liu et al., 2004; Balakrishnan and Bambara, 2013). Thus, if the difference in the polarity of the replicative helicase alleviates potentially serious problems that arise as a result of fork fusions, it might at least in part explain the difference in origin dosage.

This does not mean that the fusion of forks is unproblematic in eukaryotic cells. On the contrary, recent work has highlighted that replisome disassembly is highly choreographed and that multiple accessory proteins, such as the helicases Rrm3 and Pif1, are necessary for bringing replication to an accurate conclusion (Steinacher et al., 2012; Maric et al., 2014; Moreno et al., 2014; Dewar et al., 2015; Moreno and Gambus, 2015; Dewar and Walter, 2017; Gambus, 2017; Deegan et al., 2019), highlighting that we have only just started to understand the mechanisms and regulation of fork fusion events both in bacteria and eukaryotes.

## Author Contributions

AS, JD, OS, and CR contributed to writing the manuscript.

## Conflict of Interest

The authors declare that the research was conducted in the absence of any commercial or financial relationships that could be construed as a potential conflict of interest.
